# Post-traumatic Bone Marrow Edema of the Foot in a Child: A Case Report

**DOI:** 10.7759/cureus.108548

**Published:** 2026-05-09

**Authors:** Azucena Lirio Armas Alvarez, Angel Alois Osorio Manyari

**Affiliations:** 1 Urology, Hospital Don Benito-Villanueva de la Serena, Don Benito, ESP; 2 General Surgery, Hospital Don Benito-Villanueva de la Serena, Don Benito, ESP

**Keywords:** bone marrow edema, foot mri, foot pain, magnet therapy, physical therapy

## Abstract

Bone marrow edema in children is an uncommon condition, most often associated with trauma, and typically presents with persistent pain and difficulty walking. Conservative management, including rest and physical therapy, is commonly used.

We report the case of an 11-year-old boy with no significant past medical history who sustained a fall while playing, resulting in right foot pain. One week later, due to persistent pain and difficulty walking, he was evaluated by a traumatologist. Plain radiography showed no abnormalities, and the injury was initially managed as a sprain. As symptoms persisted after two weeks, magnetic resonance imaging (MRI) was performed, revealing bone marrow edema in the right foot. The patient was treated with anti-inflammatory medication, non-weight-bearing, and physical therapy, including magnetotherapy. He used crutches for six months. Follow-up MRI at five months demonstrated resolution of the edema. The patient subsequently resumed swimming and began walking in water, gradually overcoming his fear of weight-bearing. He progressed to ambulation with one crutch and then independently, returning to normal daily activities.

A high index of clinical suspicion is important in patients with persistent pain and gait impairment after trauma. MRI is the preferred modality for confirming the diagnosis. This case highlights the potential role of conservative management in pediatric bone marrow edema; however, given the single-case design, the observed recovery may reflect the natural course of the condition.

## Introduction

Bone marrow edema (BME) is a relatively uncommon condition in the paediatric population. It is characterized by the pathological accumulation of fluid within the bone marrow. Its aetiology may be idiopathic, ischemic, metabolic, mechanical, degenerative, or traumatic [1,2]. BME most commonly affects the weight-bearing joints, such as the ankle and foot, the knee, and the proximal femur.

In children, it often presents with nonspecific symptoms including persistent pain, discomfort exacerbated by movement, and difficulty walking, which may significantly impair daily activities [3,4]. Known risk factors for BME include trauma, corticosteroid therapy, hypercortisolism, alcohol abuse, smoking, various coagulopathies, and vitamin D deficiency [1,2,5].

BME is an important but often under-recognized cause of persistent foot pain in children, making its diagnosis challenging. Initial evaluation typically includes plain radiography, which is frequently normal and may delay diagnosis. MRI is the most sensitive modality for detecting BME and plays a key role in confirming the diagnosis and excluding other serious conditions. It is considered a safe and reliable imaging technique in children, making it especially valuable in the evaluation of persistent post-traumatic pain in this population. Given these diagnostic challenges, early recognition of BME is essential to guide appropriate management and avoid prolonged morbidity.

This case report aims to describe a pediatric case of post-traumatic bone marrow edema of the foot diagnosed by MRI and managed conservatively, highlighting the importance of clinical suspicion and appropriate imaging in children with persistent foot pain.

## Case presentation

An 11-year-old boy with no significant past medical history presented with pain in his right foot associated with difficulty walking. He had sustained a fall three days earlier while playing with friends; he was running, turned, and fell, injuring his right foot. The patient described intermittent sharp pain localized to the dorsum of the right foot at the level of the cuboid, with a visual analog scale (VAS) score of 6 at peak intensity. The pain had an acute onset and was more frequent at night. It was also described as a dull ache during weight-bearing, significantly impairing ambulation and necessitating the use of crutches. Symptoms were exacerbated by walking and partially relieved by rest and analgesics. No radiation of pain was reported. Mild swelling was noted, without associated numbness or tingling.

**Figure 1:**
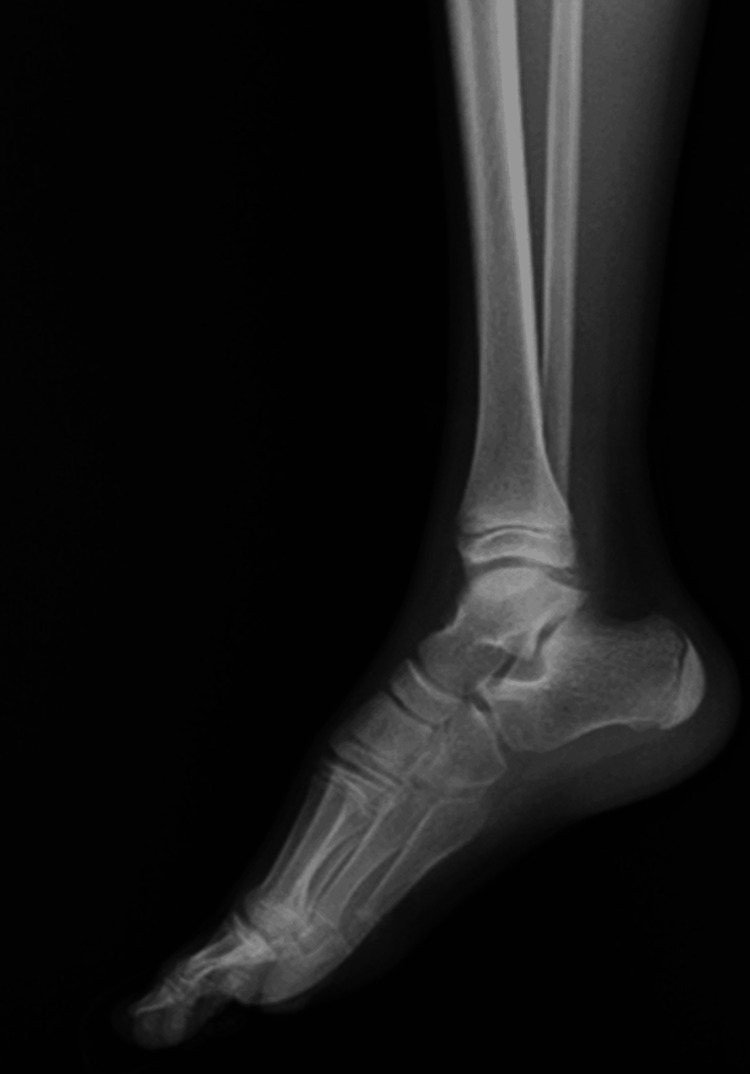
Lateral Right Foot X-ray Lateral right foot X-ray showing no abnormalities.

As the pain persisted for one week, he was evaluated by a traumatologist, who ordered an X-ray that was normal and diagnosed a sprain (Figure 1).

**Figure 2:**
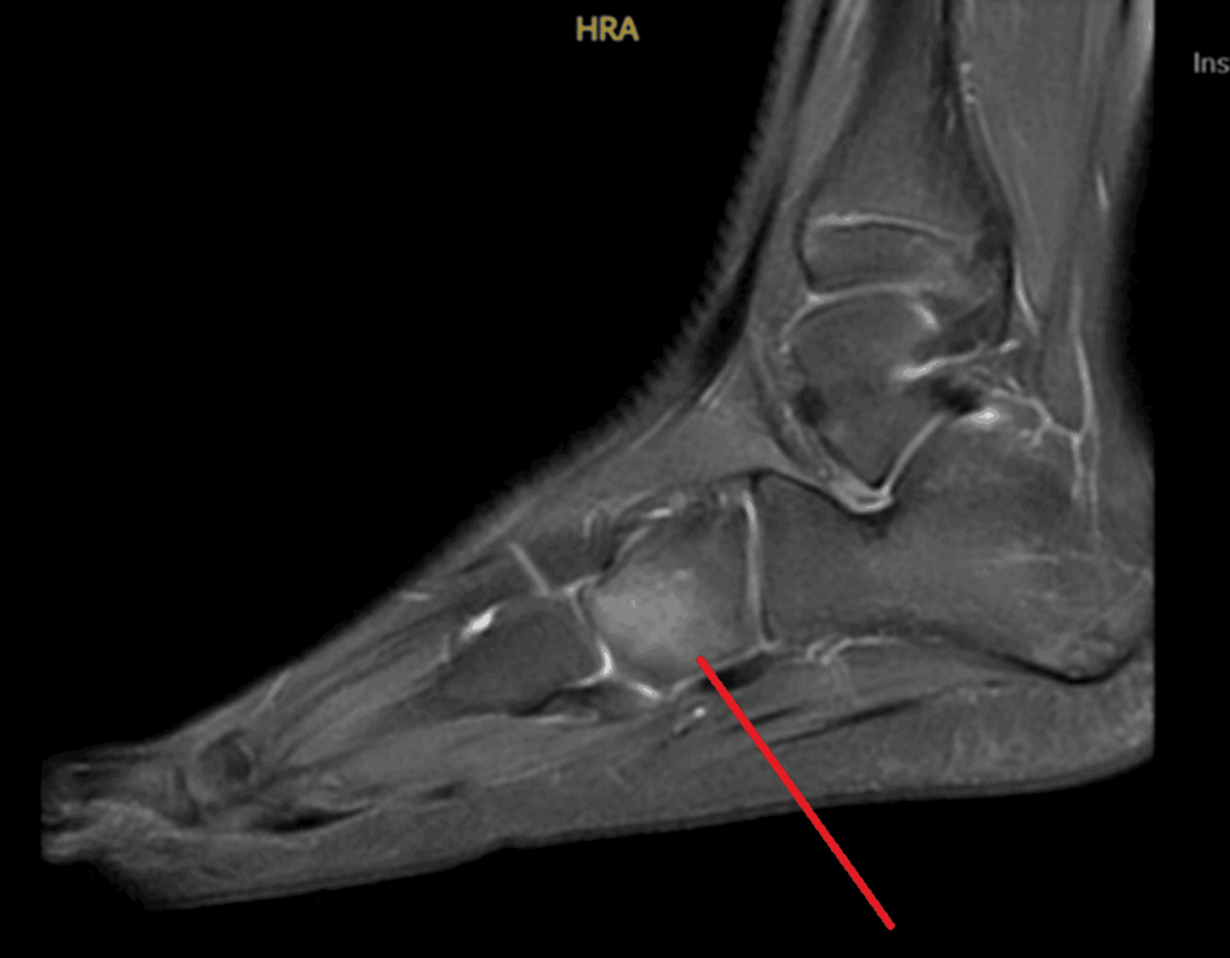
Sagittal MRI of the Right Foot Sagittal MRI demonstrates bone marrow edema in the cuboid.

**Figure 3:**
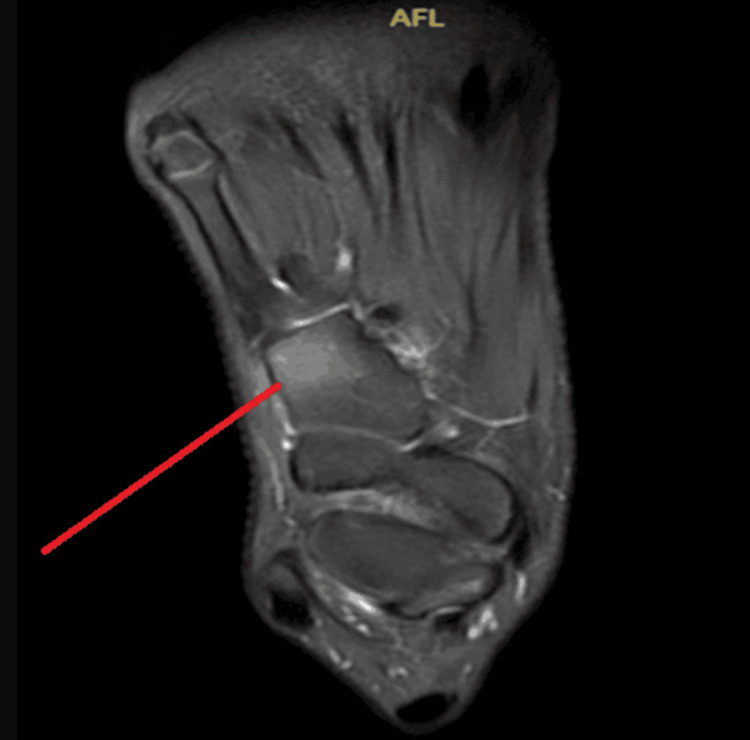
T2-Weighted Coronal MRI of the Right Foot T2-weighted coronal MRI showing bone marrow edema in the right cuboid and navicular bones.

As pain and walking impairment persisted for more than two weeks, MRI and blood tests, including vitamin D levels, were performed. MRI revealed bone marrow edema in the right foot (Figures 2, 3), and blood test results were within normal limits.

The patient was advised non-weight-bearing, use of crutches, and physical therapy, including 10 sessions of magnetotherapy, along with anti-inflammatory medication.

**Figure 4:**
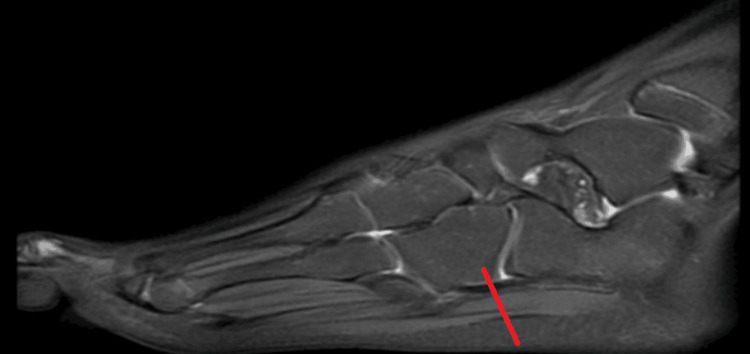
Control MRI Right Foot Control MRI shows complete resolution of bone marrow edema.

Physical therapy included activity modification and offloading with the use of crutches for six months, followed by a gradual return to weight-bearing. Therapeutic exercises focused on strengthening the surrounding muscles, correcting biomechanics, and incorporating low-impact activities such as cycling were performed daily for three months and then on alternate days for an additional two months. Manual therapy, including soft tissue and joint mobilization, followed the same schedule. Follow-up MRI at five months demonstrated resolution of the bone marrow edema (Figure 4).

Pain severity decreased, with a VAS score of 2. The patient resumed swimming pool activities and gradually began walking in the water, overcoming his fear of weight-bearing as part of hydrotherapy. He then progressed to walking with one crutch and subsequently independently, gaining confidence in ambulation. He ultimately returned to a normal, active lifestyle.

One month later, the patient reported heartburn and burping and was diagnosed with gastroesophageal reflux by a gastroenterologist, who prescribed proton pump inhibitors for eight weeks. Subsequently, his gastrointestinal symptoms improved.

## Discussion

Clinical diagnosis of bone marrow edema is challenging because its symptoms are nonspecific, such as joint pain and difficulty walking. Therefore, a high index of suspicion is required in cases of persistent joint pain and gait impairment following minor trauma, as in our case.

Bone marrow edema may progress to avascular necrosis and is considered a potential precursor to bone erosion. Its presence has been associated with an increased risk, up to sixfold, of disease progression and a more aggressive clinical course [6,7]. Therefore, early diagnosis, identification of the underlying condition, and timely initiation of treatment are essential.

Plain radiography is often normal or may show localized osteopenia [2]. Magnetic resonance imaging (MRI) is the gold standard for diagnosing bone marrow edema and allows exclusion of other serious conditions. MRI typically demonstrates patchy areas of increased signal intensity on T2-weighted and turbo inversion recovery magnitude (TIRM) sequences, with corresponding decreased signal intensity on T1-weighted images [2,7]. These signal changes may resolve over time with appropriate management. MRI is a safe imaging modality in children and should be performed when bone marrow edema is suspected.

Trauma is the most common risk factor for bone marrow edema. Microtrauma to trabecular bone leads to increased remodeling and angiogenesis, resulting in interstitial fluid accumulation within the marrow space [7]. Bone marrow edema may also occur secondary to traumatic, infectious, inflammatory, degenerative, neoplastic, ischemic, or metabolic conditions, which should be considered in the differential diagnosis, particularly in pediatric patients presenting after minor trauma [8].

Several studies have reported a correlation between bone marrow edema and vitamin D deficiency [9,10]. It has been proposed that, in vitamin D-deficient bone, microdamage may accelerate biological processes such as blood flow, cellular metabolism, and tissue remodeling, leading to a transient increase in unmineralized bone. However, other studies have found no clear causal relationship between vitamin D status, osteopenia, and bone marrow edema [8].

In the present case, vitamin D levels were within normal limits, suggesting that hypovitaminosis D was unlikely to have contributed to the development of bone marrow edema. This may indicate that mechanical factors related to trauma played a more prominent role than metabolic factors in this patient. Nevertheless, given the potential association reported in the literature and the high prevalence of vitamin D deficiency in the pediatric population, screening may be considered.

Recovery may be prolonged, with resolution of pain and return to normal ambulation typically occurring between three and 36 months [7]. A gradual return to activity is recommended. Prolonged bed rest should be avoided, as it may lead to muscle weakness and delayed recovery; however, excessive loading should also be avoided, as it may exacerbate symptoms. Patients should maintain light daily activities as tolerated [11]. The favorable outcome observed in our patient may be explained by early offloading and adherence to a structured rehabilitation program.

Treatment is not yet standardized. An initial conservative approach is recommended, including activity modification, limited weight-bearing, rest, physical therapy, and nonsteroidal anti-inflammatory drugs [1]. Physical therapy plays a central role, with goals including pain reduction, protection of affected structures, restoration of function, and safe return to weight-bearing activities. This case supports the effectiveness of a conservative, rehabilitation-focused approach, particularly in pediatric patients, where healing potential is high.

Physical therapy interventions include activity modification and offloading, therapeutic exercises focused on strengthening surrounding muscles, correcting biomechanics, and incorporating low-impact activities such as cycling, as well as proprioceptive training and gait re-education. Manual therapy and hydrotherapy may also be beneficial, improving strength, flexibility, and functional recovery.

In our case, following radiological resolution of bone marrow edema, the patient resumed swimming and initiated hydrotherapy, progressively walking in water. The buoyancy of water reduces joint loading, allowing safer mobilization and facilitating recovery.

Additional modalities such as pulsed electromagnetic field therapy or extracorporeal shock wave therapy have been explored, although evidence remains limited [11,12]. Other treatment options include pharmacological therapies (e.g., bisphosphonates, iloprost), hyperbaric oxygen therapy, and infiltrative treatments such as platelet-rich plasma or mesenchymal stem cells. In refractory cases, surgical options such as subchondroplasty or core decompression may be considered [1,11,13]. However, conservative management remains the first-line approach.

## Conclusions

A high index of clinical suspicion for bone marrow edema is important, particularly in patients presenting with persistent pain and difficulty walking after trauma. Magnetic resonance imaging remains the preferred modality for confirming the diagnosis and is considered safe in children. This case highlights the potential role of conservative management, including activity modification and physical therapy, in the clinical improvement of pediatric bone marrow edema. However, given the nature of a single case report, no definitive conclusions can be drawn regarding treatment efficacy, and the observed recovery may reflect the natural course of the condition. Further studies are required to better define optimal management strategies.
